# Secondary Hemophagocytic Lymphohistiocytosis: A Series of Three Cases

**DOI:** 10.7759/cureus.46044

**Published:** 2023-09-27

**Authors:** Rajdeep Porel, Vijay Kumar, Ketan Agarwal, Ratnadeep Biswas, Vishnu S Ojha

**Affiliations:** 1 Internal Medicine, All India Institute of Medical Sciences, Patna, IND

**Keywords:** secondary hemophagocytic lymphohistiocytosis, bone marrow, immune system diseases, antitubercular agents, cytokine release syndrome, lupus erythematosus, systemic lupus erythematosus, dengue, tuberculosis, hemophagocytic lymphohistiocytosis

## Abstract

Hemophagocytic lymphohistiocytosis (HLH) is a disease of abnormal activation of the immune system, either due to a familial cause or a sporadic cause, in relation to various triggering agents. Secondary HLH is a complication of various diseases, such as infections, malignancies, and autoimmune disorders. In our case series, we present three cases of secondary HLH with varied etiologies. Case 1 involved an 18-year-old male with a history of pulmonary tuberculosis, presenting with fever, hepatosplenomegaly, and elevated inflammatory markers. HLH was treated with steroids alongside antitubercular therapy (ATT). In case 2, a 17-year-old male presented with dengue fever, fever, hepatosplenomegaly, and elevated inflammatory markers. HLH was managed with steroids and etoposide. In case 3, a 29-year-old female with systemic lupus erythematosus (SLE) presented with fever, hepatosplenomegaly, and a positive antinuclear antibody (ANA) test. Steroid therapy was initiated for HLH. The prognosis depends on various factors. The management of such cases necessitates expeditious treatment of the underlying disease in conjunction with amelioration of the cytokine storm with the immunosuppressive agents precipitated by the secondary conditions. Once the underlying cause of the cytokine storm is treated, the lethal progression of the disease may come to a halt.

## Introduction

Hemophagocytic lymphohistiocytosis (HLH) is a disease of abnormal activation of the immune system, either due to a familial cause or a sporadic cause, in relation to various triggering agents [1]. It can be classified into two types: primary HLH and secondary HLH. Primary or familial HLH is an autosomal recessive disorder characterized by mutations in genes coding for proteins responsible for cytotoxic T cell and natural killer cell (NK cell) activation [2]. Secondary HLH is a complication of various diseases, such as infections, malignancies, and autoimmune disorders. Moreover, in both types of HLH, hypercytokinemia remains a common identity due to abnormal activation of T cells [3]. This results in a plethora of clinical manifestations. The initial clinical presentation usually starts with nonspecific constitutional symptoms and fever, with hepatosplenomegaly remaining the most common clinical finding [4]. It is also associated with seizures, bleeding manifestations, cytopenia, bone marrow suppression, etc., making it a life-threatening condition. Here, we present a case series of three patients suffering from secondary HLH due to some common etiologies (tuberculosis, dengue, and systemic lupus erythematosus (SLE)), highlighting the need for higher clinical suspicion for HLH in these diseases.

## Case presentation

Case 1

An 18-year-old male presented with a high-grade intermittent fever (104 degrees Fahrenheit) with chills and rigors for one month. The fever was not associated with day/night periodicity and was relieved after medication. There was a history of pulmonary tuberculosis at the age of three years, for which a full course of antitubercular therapy (ATT) was taken. The patient was well-oriented with an average build. Pallor was present, but icterus, clubbing, cyanosis, edema, and lymphadenopathy were absent. On palpation, tenderness in the left upper quadrant was noted. Hepatosplenomegaly was also present. Other systemic examinations were unremarkable.

Biochemical analysis revealed that the serum ferritin level was 1,650 nanograms per milliliter (ng/mL), the serum lactate dehydrogenase (LDH) level was 2,850 units per liter (U/L), the serum triglyceride level was 334 milligrams per deciliter (mg/dL), the procalcitonin level was 0.5 ng/mL, and the D-dimer level was 0.8 micrograms per milliliter (mcg/mL). The laboratory parameters of the patient on admission and at discharge are given in Table 1.

**Table 1:** Laboratory parameters of the patient on admission and at discharge

Parameter (unit)	Reference range	On admission	At discharge
Hemoglobin (grams per deciliter)	13-17	11	11.9
White blood cells (cells per cubic millimeter)	4,000-10,000	2,110	4,000
Platelet count (cells per cubic millimeter)	150,000-450,000	83,000	303,000
Alanine transaminase (units per liter)	13-40	100	125
Aspartate transaminase (units per liter)	<37	75	75
Total bilirubin (milligrams per deciliter)	0.3-1.2	0.78	0.9
C-reactive protein (milligrams per liter)	0-5	65	8

**Figure 1:**
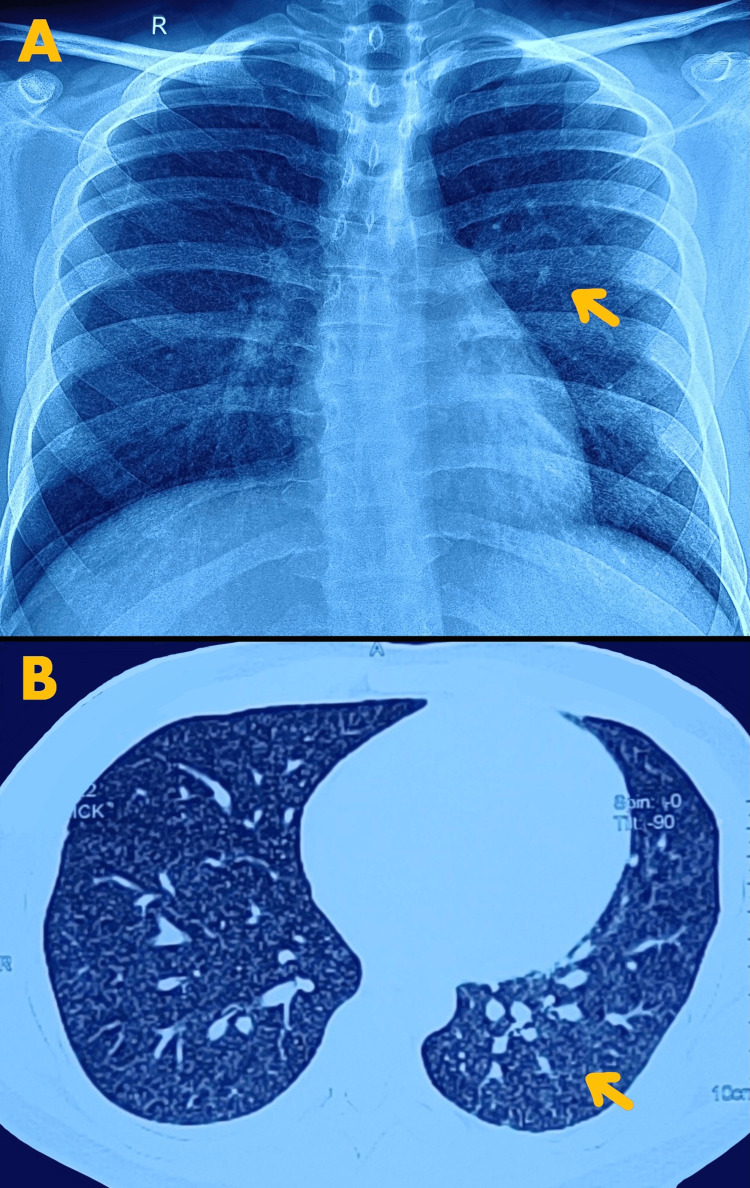
X-ray and high-resolution computed tomography images of the thorax suggestive of miliary tuberculosis A: The arrow points toward miliary mottling in the lungs on the chest radiograph. B: The arrow points toward miliary mottling and tree-in-bud appearance on high-resolution computed tomography of the thorax.

A chest X-ray showed miliary mottling in the lungs. High-resolution computed tomography (HRCT) of the thorax visualized a pattern of tree-in-bud appearance along with necrosed lymph nodes, which was suggestive of active tuberculosis infection (Figure 1).

**Figure 2:**
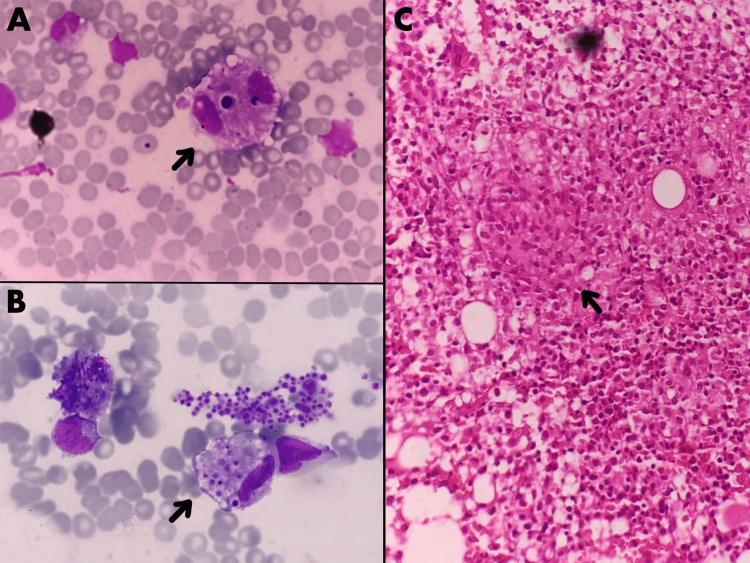
Bone marrow aspirate smear and biopsy of the patient with disseminated tuberculosis (Giemsa stain) A and B: Bone marrow aspirate smears with arrows pointing to hemophagocytic histiocytes. C: Bone marrow biopsy with the arrow showing granulomatous inflammation with epithelioid cells.

Aspirates from bronchoalveolar lavage did not show any microorganisms on the Ziehl-Neelsen stain, but a cartridge-based nucleic acid amplification test (CB-NAAT) detected the presence of *Mycobacterium tuberculosis*, which was sensitive to rifampicin. Bone marrow aspirates showed hemophagocytes with platelet and erythroid engulfment, and the bone marrow biopsy showed granulomatous inflammation with epithelioid cells (Figure 2).

Thus, a diagnosis of disseminated tuberculosis with secondary HLH was made. The patient was treated with a conventional fixed drug combination of ATT, including isoniazid, rifampicin, ethambutol, and pyrazinamide. Acetaminophen was given to control the fever. Once the diagnosis of HLH was established, steroid therapy was initiated with intravenous dexamethasone at a dose of 8 mg twice daily for one week, followed by 4 mg twice daily for one week, and then 4 mg once daily for one week, after which it was discontinued. The patient showed marked improvement over the course of the hospitalization and was then discharged in stable condition.

Case 2

A 17-year-old male presented with a high-grade intermittent fever (104 degrees Fahrenheit) with chills for two weeks. Fever was associated with a progressive increase in yellowish discoloration of the eyes and urine. There was one episode of bloody vomit and black stool. A general examination showed the presence of pallor, icterus, and bilateral pitting pedal edema. Cyanosis, clubbing, and lymphadenopathy were absent. Abdominal examination reveals tenderness in the right upper quadrant with hepatomegaly (16 centimeters (cm) of liver span) and splenomegaly (4 cm below the left costal margin). Other systemic examinations were unremarkable.

**Figure 3:**
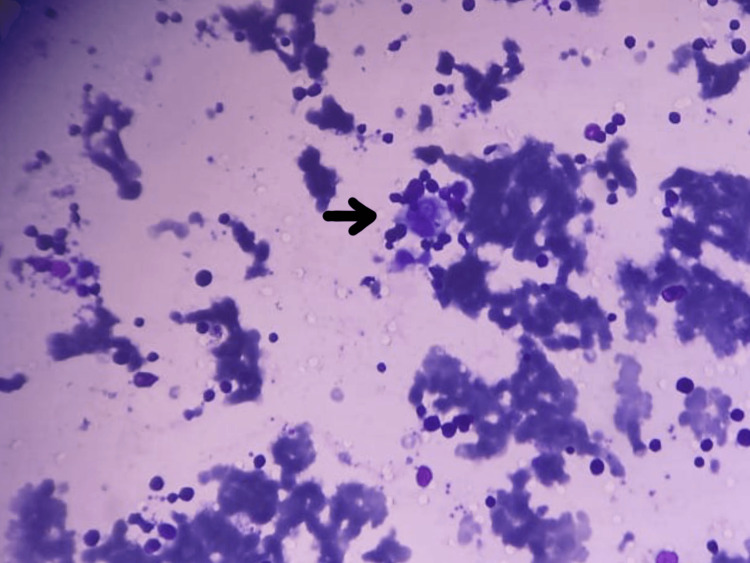
Bone marrow aspirate smear with the arrow pointing toward a hemophagocytic histiocyte in the patient with dengue (Giemsa stain)

Biochemical tests reveal that the serum ferritin level was 1,680 ng/mL, the LDH level was 8,690 U/L, and the serum triglyceride level was 290 mg/dL. Markers of dengue fever, such as antibodies against the dengue nonstructural protein one (NS1) antigen and immunoglobulin M (IgM) antibodies, were positive. Hemophagocytes were visualized in bone marrow aspirates (Figure 3). Thus, a diagnosis of dengue infection leading to secondary HLH was made.

The laboratory parameters of the patient on admission, before the initiation of therapy, and at discharge are given in Table 2.

**Table 2:** Laboratory parameters of the patient on admission, before the initiation of therapy, and at discharge

Parameter (unit)	Reference range	On admission	Before the initiation of therapy	At discharge
Hemoglobin (grams per deciliter)	13-17	7.5	5.3	7.3
White blood cells (cells per cubic millimeter)	4,000-10,000	870	3,900	5,680
Platelet count (cells per cubic millimeter)	150,000-450,000	45,000	80,000	165,000
Alanine transaminase (units per liter)	13-40	1,200	872	150
Aspartate transaminase (units per liter)	<37	275	1,886	260
Total bilirubin (milligrams per deciliter)	0.3-1.2	4.6	16.6	8.5
Albumin (grams per deciliter)	3.4-4.8	2.4	2.1	2.9

The patient was empirically started on piperacillin/tazobactam, and packed red blood cells (RBC), platelets, and fresh frozen plasma were transfused judiciously. Steroid therapy with dexamethasone and etoposide was also concurrently initiated following the HLH-94 protocol. The patient showed significant improvement and was discharged in stable condition.

Case 3

A 29-year-old female came with complaints of a continuous fever (103 degrees Fahrenheit) with chills for two months. During the patient’s hospitalization, she began experiencing symptoms such as headache, confusion, and restlessness combined with incomprehensible speech. There was a history of non-bilious vomiting, reduced appetite, and weight loss. A general examination showed pallor, but icterus, clubbing, cyanosis, and lymphadenopathy were absent. On palpation, there was the presence of hepatomegaly (the liver span was 17 cm) and splenomegaly (2 cm below the costal margin). The rest of the systemic examination was unremarkable.

**Figure 4:**
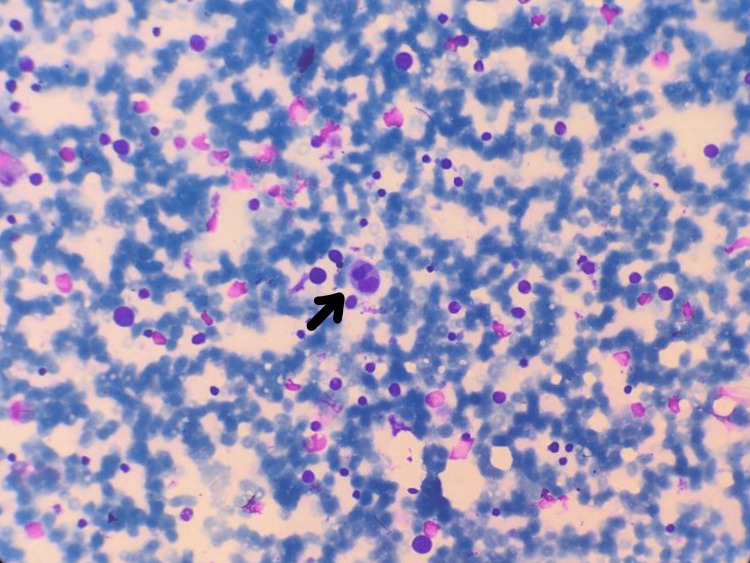
Bone marrow aspirate smear with the arrow pointing to a hemophagocytic histiocyte in the patient with systemic lupus erythematosus (Giemsa stain)

Biochemical analysis shows serum ferritin at 1,100 ng/mL, serum triglyceride at 395 mg/dL, fibrinogen at 115 mg/dL, and LDH at 2,080 U/L. Contrast-enhanced computed tomography (CECT) of the thorax revealed the presence of a solitary lymph node in the right hilar region of the body. Additionally, there was a slight accumulation of fluid in the pleural cavity on the right side, and the CECT of the abdomen showed an enlargement of the liver and spleen with mild ascites, which was suggestive of serositis. The screening test for antinuclear antibodies (ANA) using indirect immunofluorescence (IIF) on HEp-2 cells yielded a positive result with an estimated titer of 1:100. The diagnosis of systemic lupus erythematosus (SLE) was established based on the Systemic Lupus International Collaborating Clinics (SLICC) criteria, considering the presence of hemolytic anemia, thrombocytopenia, leukopenia, neurological manifestations, serositis, and a positive ANA screen. Bone marrow aspirates showed the presence of hemophagocytes (Figure 4).

The laboratory parameters of the patient on admission are given in Table 3.

**Table 3:** Laboratory parameters of the patient on admission

Parameter (unit)	Reference range	On admission
Hemoglobin (grams per deciliter)	13-17	8.3
White blood cells (cells per cubic millimeter)	4,000-10,000	2,800
Platelet count (cells per cubic millimeter)	150,000-450,000	95,000
Alanine transaminase (units per liter)	13-40	77
Aspartate transaminase (units per liter)	<37	153
Total bilirubin (milligrams per deciliter)	0.3-1.2	0.87
Albumin (grams per deciliter)	3.4-4.8	2.74

The patient was started on empirical broad-spectrum antibiotics. Once the diagnosis of SLE with secondary HLH was made, the patient was initiated on steroid treatment with intravenous dexamethasone 8 mg twice daily. Quetiapine and haloperidol were added for psychotic behavior. The patient was shifted to the intensive care unit due to a low Glasgow Coma Scale (GCS) score. Non-contrast computed tomography of the head was unremarkable. Elective intubation was done. While in the intensive care unit, the patient developed ventilator-associated pneumonia and septic shock, for which inotropes and colistin were added as the culture and sensitivity report showed growth of *Acinetobacter baumannii* sensitive to colistin only, yet the condition of the patient deteriorated, and unfortunately, the patient suffered a cardiac arrest and could not be revived.

## Discussion

HLH is a hyperinflammatory disorder characterized by hypercytokinemia. The key pathognomonic feature of HLH is the persistence and activation of cytotoxic T cells (CD8+ T cells). In primary HLH, the main contributing factor is a genetic abnormality related to the process of cytolysis. In our cases, the causes of HLH were secondary, i.e., due to underlying diseases. It is thought that the continuous secretion of interferon-gamma by activated CD8+ T cells triggers the activation of tissue macrophages and monocytes, leading to the release of inflammatory agents such as interleukin (IL)-18, IL-6, and IL-12. IL-18 and IL-12 may further boost the production of interferon-gamma by CD8+ T cells [5].

The clinical picture of the disease is mainly dependent on the underlying trigger but usually presents nonspecifically with fever, skin rash, neurological symptoms, and hepatosplenomegaly [6]. In all of our cases, hepatosplenomegaly was noted. The reason behind this may be inflammation in the portal-sinusoidal region. The macrophages exhibit the production of interferon-gamma, tumor necrosis factor-alpha, and IL-2 and increased expression of granulocytic monocytic colony-stimulating factor receptors, leading to their proliferation and increased size of the liver and spleen [7].

The nonspecific presentation and lack of specific signs or symptoms render its diagnosis challenging. In the abovementioned cases of HLH, the diagnosis was made based on the HLH-2004 guidelines proposed by Henter et al. [8]. To establish a diagnosis of hemophagocytic lymphohistiocytosis, it is necessary to meet a minimum of five out of eight specified criteria. These criteria include fever, enlargement of the spleen, a decrease in blood cells in more than two cell lines, elevated levels of triglycerides or a decrease in fibrinogen, the identification of hemophagocytes through bone biopsy, the detection of hemophagocytes in the lymph nodes or spleen, the reduced or absent activity of natural killer cells, and an increase in soluble interleukin-2 receptor subunit alpha (CD25) receptor levels. Compliance with these diagnostic criteria is crucial for the accurate diagnosis of HLH [8], and all three cases satisfied these criteria.

The pathophysiology behind the laboratory changes in HLH is linked to the cytokine response. Increased levels of tumor necrosis factor-alpha and interferon-gamma accompanied by hemophagocytosis are responsible for reduced cell numbers in various cell lines [9]. Hyperferritinemia is explained by the anti-inflammatory activity of macrophages, as the known mechanism for heme phagocytosis by macrophages is through the cluster of differentiation 163 (CD163) receptor, which ultimately increases iron stores in the body, causing hyperferritinemia [10].

However, the reason behind the increased levels of bilirubin in the blood seen in case 2 was probably the development of cholestasis due to the damage that lymphocytes and lymphohistiocytes cause to the biliary ductules. There are also raised levels of triglycerides in all the HLH cases, which can be attributed to the impaired activity of lipoprotein lipase caused by various cytokines (tumor necrosis factor-alpha, IL-1, and IL-6) [7].

Since HLH has been an inflammatory endpoint in all three cases, treatment of the primary disease becomes crucial. For instance, in the first case, with the start of ATT, there was a dramatic improvement in the patient’s condition. It is also possible that the attainment of remission of the underlying disease can improve the efficacy of immunosuppressive therapy targeting the treatment of HLH [11].

Secondary HLH is a potentially fatal disease with more than 50% mortality. The survival of a patient depends on various predisposing factors. Based on the study conducted by Kumakura et al. [12], autoimmune conditions that lead to HLH have a more favorable prognosis. However, in contrast to this finding, the third patient (who had SLE and secondary HLH) experienced a significantly severe disease course. The possible explanation for this may be the presence of poor prognostic markers, as indicated by Brito-Zerón et al. [13], such as a platelet count of fewer than 100,000 cells per cubic millimeter, a total leukocyte count of fewer than 4,000 cells per cubic millimeter, and an additional trigger such as a bacterial infection. Additional risk factors that may be sought to contribute to the outcome may be very high levels of LDH (more than 1000 U/L) and low levels of fibrinogen (less than 150 mg/dL) [14].

## Conclusions

In conclusion, a diverse range of symptoms and signs may be observed in cases of secondary HLH. Thus, early diagnosis of HLH becomes crucial in different contexts with secondary etiologies. The management of such cases necessitates expeditious treatment of the underlying disease in conjunction with amelioration of the cytokine storm with the immunosuppressive agents precipitated by the secondary conditions. Once the underlying cause of the cytokine storm is treated, the lethal progression of the disease comes to an end.

## References

[1] Jordan MB, Allen CE, Weitzman S, Filipovich AH, McClain KL (2011). How I treat hemophagocytic lymphohistiocytosis. Blood.

[2] Wysocki CA (2017). Comparing hemophagocytic lymphohistiocytosis in pediatric and adult patients. Curr Opin Allergy Clin Immunol.

[3] Morimoto A, Nakazawa Y, Ishii E (2016). Hemophagocytic lymphohistiocytosis: pathogenesis, diagnosis, and management. Pediatr Int.

[4] Memon F, Ahmed J, Malik F, Ahmad J, Memon DA (2020). Adult-onset primary hemophagocytic lymphohistiocytosis: reporting a rare case with review of literature. Cureus.

[5] Griffin G, Shenoi S, Hughes GC (2023). Hemophagocytic lymphohistiocytosis: an update on pathogenesis, diagnosis, and therapy. Best Pract Res Clin Rheumatol.

[6] Fatima Z, Khan A, Tariq U, Sohail MS (2018). Hemophagocytic lymphohistiocytosis: a case series. Cureus.

[7] Padhi S, Sarangi R, Patra S, Samal SC (2019). Hepatic involvement in hemophagocytic lymphohistiocytosis. Hepatitis A and other associated hepatobiliary diseases.

[8] Henter JI, Horne A, Aricó M (2007). HLH-2004: Diagnostic and therapeutic guidelines for hemophagocytic lymphohistiocytosis. Pediatr Blood Cancer.

[9] George MR (2014). Hemophagocytic lymphohistiocytosis: review of etiologies and management. J Blood Med.

[10] Janka GE (2007). Familial and acquired hemophagocytic lymphohistiocytosis. Eur J Pediatr.

[11] Karlsson T (2015). Secondary haemophagocytic lymphohistiocytosis: experience from the Uppsala University Hospital. Ups J Med Sci.

[12] Kumakura S, Murakawa Y (2014). Clinical characteristics and treatment outcomes of autoimmune-associated hemophagocytic syndrome in adults. Arthritis Rheumatol.

[13] Brito-Zerón P, Kostov B, Moral-Moral P (2018). Prognostic factors of death in 151 adults with hemophagocytic syndrome: etiopathogenically driven analysis. Mayo Clin Proc Innov Qual Outcomes.

[14] Li F, Yang Y, Jin F (2015). Clinical characteristics and prognostic factors of adult hemophagocytic syndrome patients: a retrospective study of increasing awareness of a disease from a single-center in China. Orphanet J Rare Dis.

